# SETD1A promotes the proliferation and glycolysis of nasopharyngeal carcinoma cells by activating the PI3K/Akt pathway

**DOI:** 10.1515/med-2022-0586

**Published:** 2022-11-22

**Authors:** Jianyi Huang, Jinshu Fang, Xiao Xu, Xueshen Qian, Xia Zhang

**Affiliations:** Department of Classics of Traditional Chinese Medicine, Taizhou Hospital of Traditional Chinese Medicine, Taizhou, Jiangsu, 225300, China; Department of Oncology, Taizhou Hospital of Traditional Chinese Medicine, Taizhou, Jiangsu, 225300, China; Department of Clinical Laboratory, Taizhou Hospital of Traditional Chinese Medicine, Taizhou, Jianshu, 22530, China; Department of Otorhinolaryngology, Changzhou Second People’s Hospital, No. 1 29, Xinglong Lane, Tianning District, Changzhou, Jiangsu, 213003, China

**Keywords:** SETD1A, nasopharyngeal carcinoma, glycolysis, proliferation, PI3K/AKT

## Abstract

Nasopharyngeal carcinoma is one of the common malignant tumors that the pathogenesis has not yet been completely defined. SETD1A (histone lysine methyltransferase SET domain-containing 1A) is related to the occurrence of various cancers. However, the role of SETD1A in nasopharyngeal carcinoma remains unclear. The SETD1A overexpression vector, si-NC, si-SETD1A#1, and si-SETD1A#2 were transfected into nasopharyngeal carcinoma cells to overexpress or knockdown SETD1A expression. The assay of biofunction was used to explore the role of SETD1A in nasopharyngeal carcinoma cells. The assay of glucose uptake, lactate release, ATP level, western blot, cell proliferation, and cellular apoptosis analysis were performed to investigate the potential mechanism of SETD1A regulation in nasopharyngeal carcinoma. This study was the first to show that SETD1A was upregulated in nasopharyngeal carcinoma cells and the overexpression of SETD1A significantly promoted the cell proliferation and glycolysis and suppressed the cellular apoptosis. Moreover, SETD1A enhances aerobic glycolysis and cell biological function of nasopharyngeal carcinoma cells via PI3K/AKT signaling pathway. SETD1A induced PI3K/AKT activation and subsequently prevented cellular apoptosis. In conclusion, this study identified overexpressed SETD1A as a positive regulator of proliferation that induced nasopharyngeal carcinoma cells’ aerobic glycolysis via PI3K/AKT signaling activation in vitro. This study laid a strong foundation for unveiling the precise anticancer mechanism of SETD1A. The SETD1A may become a novel biomarker for further inhibitor design to obstruct the PI3K/AKT-dependent nasopharyngeal carcinoma progression.

## 1 Introduction

Nasopharyngeal carcinoma originates from the epithelial lining of the nasopharynx and is a highly aggressive head and neck cancer [1,2]. The 5-year survival rate of nasopharyngeal carcinoma patients has significantly raised due to advances in medical treatment techniques [3]. However, since most nasopharyngeal carcinoma patients are not diagnosed until stage III or IV, 30% of nasopharyngeal carcinoma patients will finally have distant metastasis and/or recurrence [4,5]. Therefore, identifying novel therapeutic targets and predictive biomarkers is important to ameliorate clinical outcomes for nasopharyngeal carcinoma patients.

The aerobic glycolysis (Warburg effect) is one of the critical hallmarks of cancer. The Warburg effect is defined as that cancer cells rely heavily on glycolysis for energy metabolism and preferential production of lactate [6,7]. Therefore, the aerobic glycolysis brings large energy to cancer cells, different from normal cells in cancer cells, glucose is not metabolized in mitochondria by oxidative phosphorylation but is converted to lactate [8]. Phosphatidylinositol 3-kinase (PI3K) and protein kinase B (AKT) are often over-activated in cancer cells and have an essential role in the aerobic glycolysis regulation in cancer cells [9,10]. After the PI3K/AKT signaling pathway is activated, it can directly promote the transition of aerobic glycolysis, making cancer cells more dependent on glucose consumption to produce lactate.

SETD1A (histone lysine methyltransferase SET domain-containing 1A) is involved in the epigenetic regulation of transcription. It is the major catalytic component of a multiprotein complex that methylates lysine 4 of histone H3 [11,12]. SETD1A encodes a subunit of the human Set/COMPASS complex (complex of proteins associating with Set1) and it might regulate multiple critical oncogenes in lung cancer via global H3K4me3 levels [13]. SETD1A plays a role in the occurrence of various cancers. The expression of SETD1A is significantly improved in lung cancer tissues [13,14]. On the contrary, the knockdown of SETD1A can suppress the proliferation of cancer cells. In addition, the expression of SETD1A was obviously enhanced in hepatocellular carcinoma cell lines and tumor tissues and SETD1A knockdown enhanced sorafenib-induced proliferation inhibition and cellular apoptosis [15]. Previous studies found that overexpression of SETD1A induced gastric cancer cell proliferation, while suppression of SETD1A attenuated the cell viability of gastric cancer cells [16]. In addition, downregulation of SETD1A decreased lactate production and glucose uptake, while inhibited glycolysis by reducing glycolytic genes’ expression such as lactate dehydrogenase A (LDHA), monocarboxylate transporter 4, pyruvate kinase M 2, glucose transporter 1 (GLUT1), and phosphofructokinase 2 [16]. Recent studies have also reported that upregulation of SETD1A can activate the PI3K/Akt pathway and promote breast cancer progression [17].

According to the TCGA database, SETD1A is highly expressed in head and neck cancer, but the role of SETD1A in nasopharyngeal carcinoma is less studied, and the related mechanism is still unclear. The purpose of this research was to explore the specific biological function and the potential mechanism of SETD1A in nasopharyngeal carcinoma.

## 2 Methods

### 2.1 Cell culture

Nasopharyngeal carcinoma cell lines (C666-1, SUNE-1, HNE-1, and 6-10b) and human nasopharyngeal epithelial cell line Np69 were obtained from the Cell Bank of Type Culture Collection of Chinese Academy of Sciences (Shanghai, China). All cells were maintained in Dulbecco’s modified Eagle’s medium (DMEM, Invitrogen, USA) containing 10% fetal bovine serum and 1% penicillin–streptomycin (Gibco) at 37°C with 5% CO_2_. When cell confluence reached 60–70%, si-NC, si-SETD1A#1, si-SETD1A#2, vector, or OE-SETD1A were transfected into C666-1 and HNE-1 cells using Lipofectamine 3000 according to the reference instructions. After 48 h, cells were collected for further experiments. To clarify the potential mechanism constituting by which SETD1A promotes the progression of nasopharyngeal carcinoma, the PI3K inhibitor LY294002 (L9908, Sigma) was performed to co-treatment with OE-SETD1A in C666-1 and HNE-1 cells. The cells were cultured in serum-free medium for 24 h and pretreated with 10 µM LY294002 before further experiments.

### 2.2 qPCR analysis

The RNA was obtained from Np69, C666-1, SUNE-1, HNE-1, and 6-10b cells using TRIzol method (Invitrogen, USA) according to the manufacturer’s instructions. The PrimeScript RT Reagent Kit (Takarabio, Japan) was used to synthesize cDNA. qRT-PCR was performed on an real-time PCR system (Bio-Rad Laboratories, USA) and detected by using SYBR Green PCR Master Mix (Thermo Fisher, USA).

The primers were as follows:

SETD1A:

forward: 5′-TTGCCATGTCAGGTCCAAAAA-3′;

reverse: 5′-GTACTTACGGCACATATCCTTC-3′,

GAPDH:

forward: 5′-TCTTTTGCGT CGCCAGCCGAG-3′

reverse: 5′-TGACCAGGCG CCCAATACGAC-3′.

### 2.3 Western blot

The total protein from Np69, C666-1, SUNE-1, HNE-1, and 6-10b cells was harvested using RIPA buffer and centrifuging at 13,000 × *g* at 4°C for 15 min. Twenty-five micrograms of protein samples were separated using the 10% sodium dodecyl sulfate polyacrylamide gel electrophoresis gel and the nitrocellulose membranes (Sigma-Aldrich, USA) were used to transfer the proteins. The primary antibody against SETD1A (1:3,000, Thermo Fisher, MA5-26764), GLUT1 (1:5,000, Cell Signaling Technology, E4S6I), HK2 (1:3,000, Cell Signaling Technology, C64G5), LDHA (1:2,000, Santa Cruz Biotechnology, C4B5), Bax (1:2,000, Cell Signaling Technology, # 2774), Bcl-2 (1:1,000, Abcam, ab196495), PI3K (1:8,000, Cell Signaling Technology, # 4292), p-PI3K (1:3,000, Cell Signaling Technology, # 4228), AKT (1:8,000, Abcam, ab8805), p-AKT (1:3,000, Abcam, ab81283), and β-actin (1:10,000, Abcam, ab8227) overnight at 4°C. The membrane was cultured with horseradish peroxidase‑labeled secondary antibody at 37°C for 1 h, and the band of proteins was detected by the enhanced chemiluminescence (Sigma-Aldrich). β-Actin was used as the internal control.

### 2.4 Clone formation assay

The C666-1 and HNE-1 cells under different treatments were seeded into six-well plates (500 cells per well). The cells were treated with sorafenib (10 μM) for 2 weeks. The sorafenib treatment was maintained and the culture medium was changed every 2 days. After 2 weeks, the cells were fixed and stained with 0.1% crystal violet. The cell clones were counted.

### 2.5 EDU (cell proliferation) 5-ethynyl-2′-deoxyuridine (EdU)

Cultured C666-1 and HNE-1 cells in the supernatant were centrifuged and plated on collagen-coated coverslips in a DMEM culture medium containing 10% fetal calf serum. Then, cells were stained by Click-iTTM EdU imaging kit (Invitrogen, CA) according to the manufacturer’s protocol for 12 h. The EdU-positive cells were measured under a fluorescence microscope.

### 2.6 ATP measurement

The cellular expression levels of ATP from treated C666-1 and HNE-1 cells were quantitatively assessed using the Cellular ADP/ATP‐Glo™ Assay (Promega AG) according to the manufacturer’s protocol. Values were normalized to the total protein content.

### 2.7 Glucose and lactate measurement

The cellular expression levels of glucose and lactate in the supernatant from treated C666-1 and HNE-1 cells were quantitatively assessed using the enzyme-linked immunosorbent assay kits according to the manufacturer’s protocol (Abcam) at room temperature.

### 2.8 Cell apoptosis assay

To investigate the cellular apoptosis in C666-1 and HNE-1 cells, the flow cytometry Annexin V-FITC/PI apoptosis detection kit was used. The cells were harvested and resuspended at a concentration of 10^6^ cells/mL. FITC-Annexin V and PI were added and kept on ice in the dark for 15 min. The flow cytometry was used to measure the apoptotic cells’ ratio.

### 2.9 Statistics

All experiments were obtained in triplicate and analyzed using GraphPad Prism Software 6.0 (GraphPad Software, La Jolla, USA). All data are presented as the mean ± standard error of the mean (SEM). One-way analysis of variance, and *t*-tests were used to make a comparison. A statistically significant difference was defined as a *p* < 0.05.

## 3 Results

### 3.1 SETD1A was upregulated in nasopharyngeal carcinoma cells

**Figure 1:**
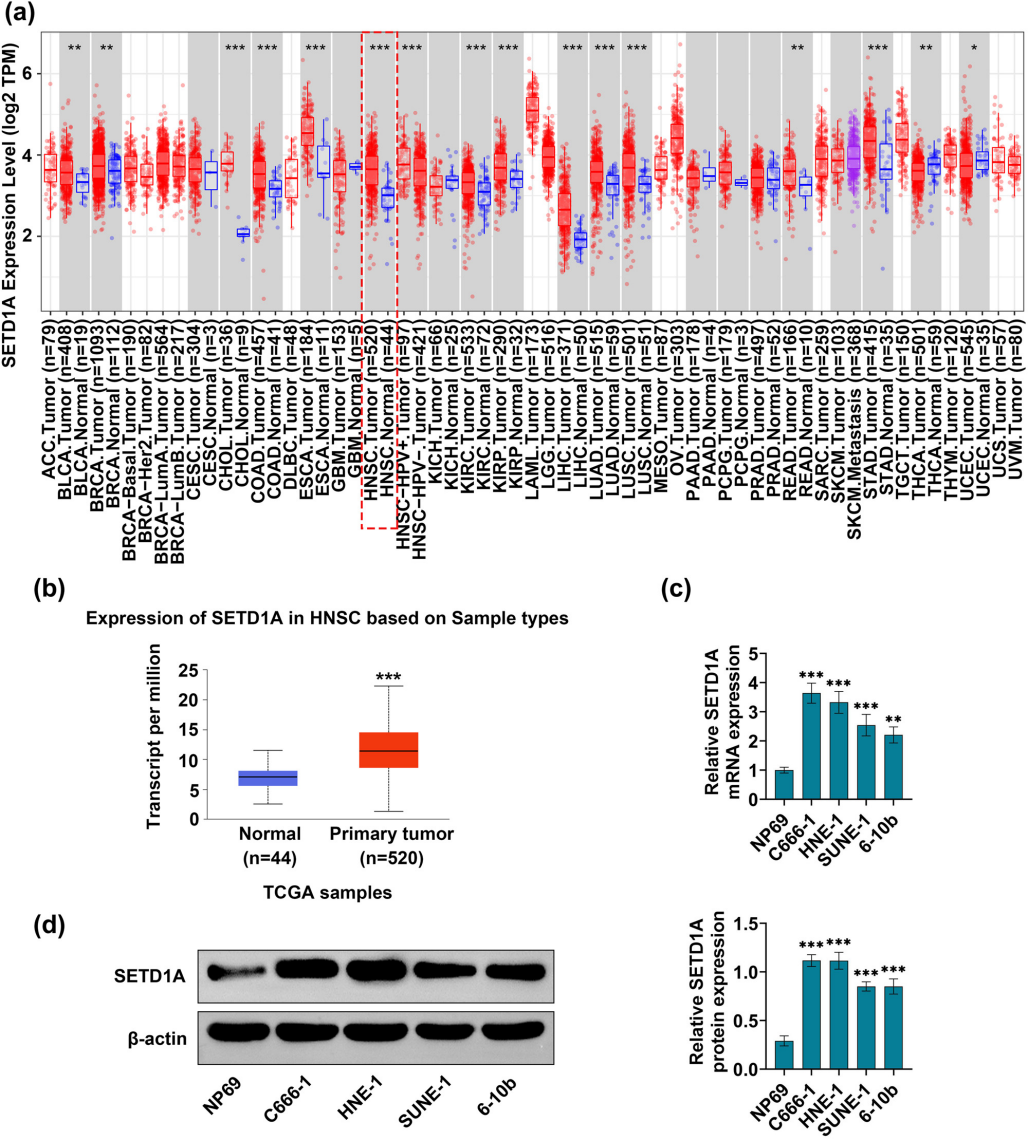
SETD1A is upregulated in nasopharyngeal carcinoma cells. (a and b) Analysis of the expression of SETD1A in normal (*n* = 44) and GC (*n* = 520) specimens from the TCGA dataset obtained by GEPIA. (c) RT-qPCR was carried out to measure mRNA expression levels of SETD1A in Np69, C666-1, SUNE-1, HNE-1, and 6-10b cells. (d) Western blot was used to explore the protein expression level of SETD1A. **p < 0.01 vs Np69. ***p < 0.005 vs Np69. Data are expressed as mean ± SEM.

To investigate whether SETD1A plays a pivotal role in nasopharyngeal carcinoma, the most authoritative database in the cancer-TCGA database was used to compare the difference in SETD1A expression in normal and nasopharyngeal carcinoma patient’s tissue. The results from cancer-TCGA database analysis identified that SETD1A was upregulated in nasopharyngeal carcinoma compared to normal tissue (Figure 1a and b). To determine whether nasopharyngeal carcinoma cells have molecular changes in SETD1A, the present study compared the difference in the expression of SETD1A in nasopharyngeal carcinoma cell lines (C666-1, SUNE-1, HNE-1, and 6-10b) and human nasopharyngeal epithelial cell line Np69 by RT-qPCR and western blotting analysis, respectively. RT-qPCR analysis revealed that the mRNA expression level of SETD1A was remarkably enhanced in the C666-1, SUNE-1, HNE-1, and 6-10b cell lines, compared with that in the human nasopharyngeal epithelial cell line (Figure 1c). Moreover, the level of SETD1A quantified by western blot assay was also presented a similar trend of variation (Figure 1d). These findings suggested that SETD1A was overexpressed in nasopharyngeal carcinoma cells.

### 3.2 SETD1A promotes the proliferation of nasopharyngeal carcinoma cells

**Figure 2:**
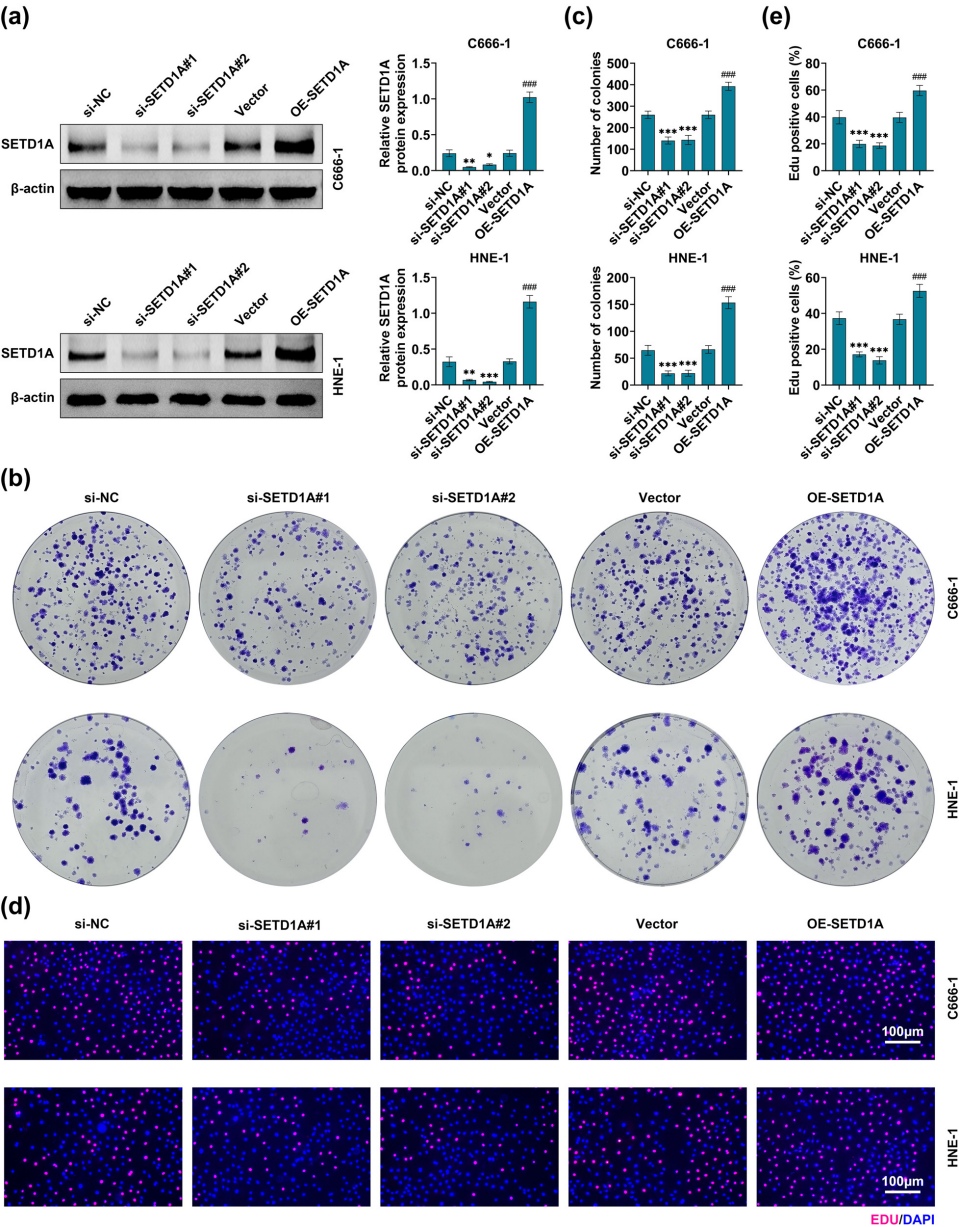
Effects of SETD1A on nasopharyngeal carcinoma cells’ proliferation. (a) After si-NC, si-SETD1A#1, si-SETD1A#2, vector, or OE-SETD1A transfected, the expression levels of SETD1A in C666-1 and HNE-1 cells were determined by western blotting. β-Actin was used as an internal control. (b and c) The colony-forming assay was used to measure the effects of SETD1A expression on cell proliferation. (d and e) The EdU staining was used to determine the effects of SETD1A expression on cell proliferation. *p < 0.05 vs si-NC. **p < 0.01 vs si-NC. ***p < 0.005 vs si-NC. ^###^ *p* < 0.005 vs vector. Data are expressed as mean ± SEM.

According to the above experimental results, we selected two cell lines, C666-1 and HNE-1, with higher expression levels of SETD1A for subsequent experiments. To determine the effect of SETD1A on the biological function of nasopharyngeal carcinoma cells, the cells were transfected with si-NC, si-SETD1A#1, si-SETD1A#2, vector, or OE-SETD1A. The western blot results showed that SETD1A was significantly inhibited after si-SETD1A#1 and si-SETD1A#2 transfected; conversely, the SETD1A expression was obviously increased while OE-SETD1A transfected (Figure 2a). The clone formation assay and EdU staining were employed to examine the cell proliferation. The clone formation assay revealed that knockdown of SETD1A remarkably suppressed the clone formation abilities in C666-1 and HNE-1 cell lines compared to the si-NC group (Figure 2b and c). Next, the EdU staining results showed that the number of EdU-positive cells was obviously reduced after SETD1A knockdown (Figure 2d and e). By contrast, upregulated SETD1A significantly enhanced the cell proliferation and clone formation abilities in C666-1 and HNE-1 cell lines compared to the vector group (Figure 2b–e). These data revealed that the expression of SETD1A enhanced cell proliferation in nasopharyngeal carcinoma cell lines.

### 3.3 SETD1A enhances glycolysis of nasopharyngeal carcinoma cells

**Figure 3:**
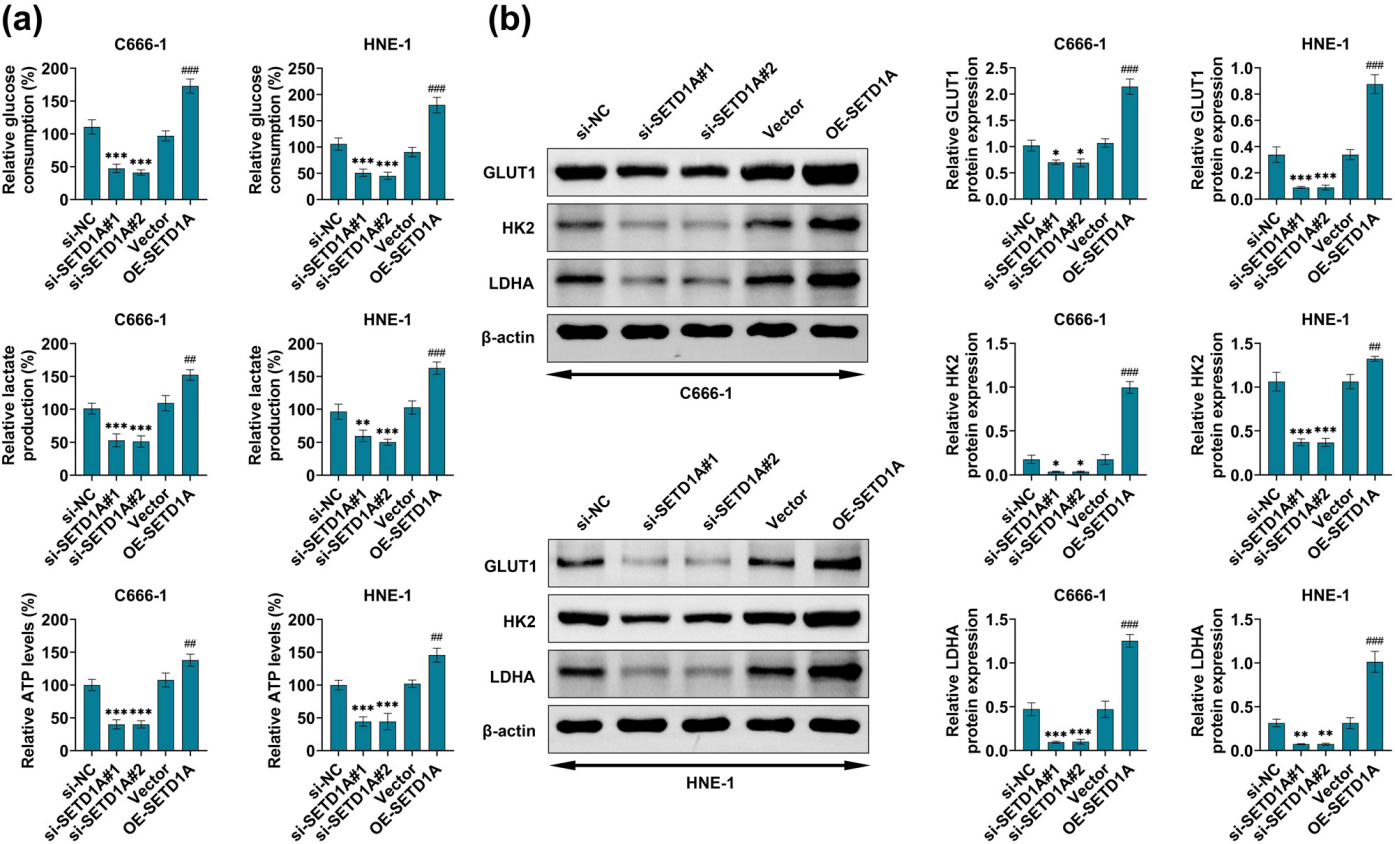
The effects of SETD1A overexpression or knockdown on Warburg effect in nasopharyngeal carcinoma cells. (a) Detection of glucose consumption, lactate production, and ATP levels in C666-1 and HNE-1 cells using commercial kits. (b) Western blot was used to explore the protein expression level of GLUT1, HK2, and LDHA. *p < 0.05 vs si-NC. **p < 0.01 vs si-NC. ***p < 0.005 vs si-NC. ^##^ *p* < 0.01 vs vector. ^###^ *p* < 0.005 vs vector. Data are expressed as mean ± SEM.

To investigate the mechanisms of glycolysis by which SETD1A regulated, the present study examined the essential enzymes and metabolic parameters involved in glucose metabolism in C666-1 and HNE-1 cells’ upregulation or downregulation of SETD1A. The data revealed that the cellular glucose uptake, cellular ATP levels, and lactate production in a culture medium were remarkably decreased after the knockdown of SETD1A in C666-1 and HNE-1 cells (Figure 3a). Conversely, overexpression of SETD1A in C666-1 and HNE-1 cells led to reversed effects on the above metabolic parameters (Figure 3a). Moreover, the western blot was used to confirm the results; SETD1A knockdown significantly reduced the expression of LDHA, GLUT1, and HK2 (Figure 3b). Meanwhile, the protein expression of GLUT1, HK2, and LDHA was obviously enhanced in C666-1 and HNE-1 cells overexpressing SETD1A (Figure 3b). Together, these data suggest that SETD1A enhances glucose metabolism by upregulating the expression of glycolytic-related proteins.

### 3.4 SETD1A suppresses the apoptosis of nasopharyngeal carcinoma cells

**Figure 4:**
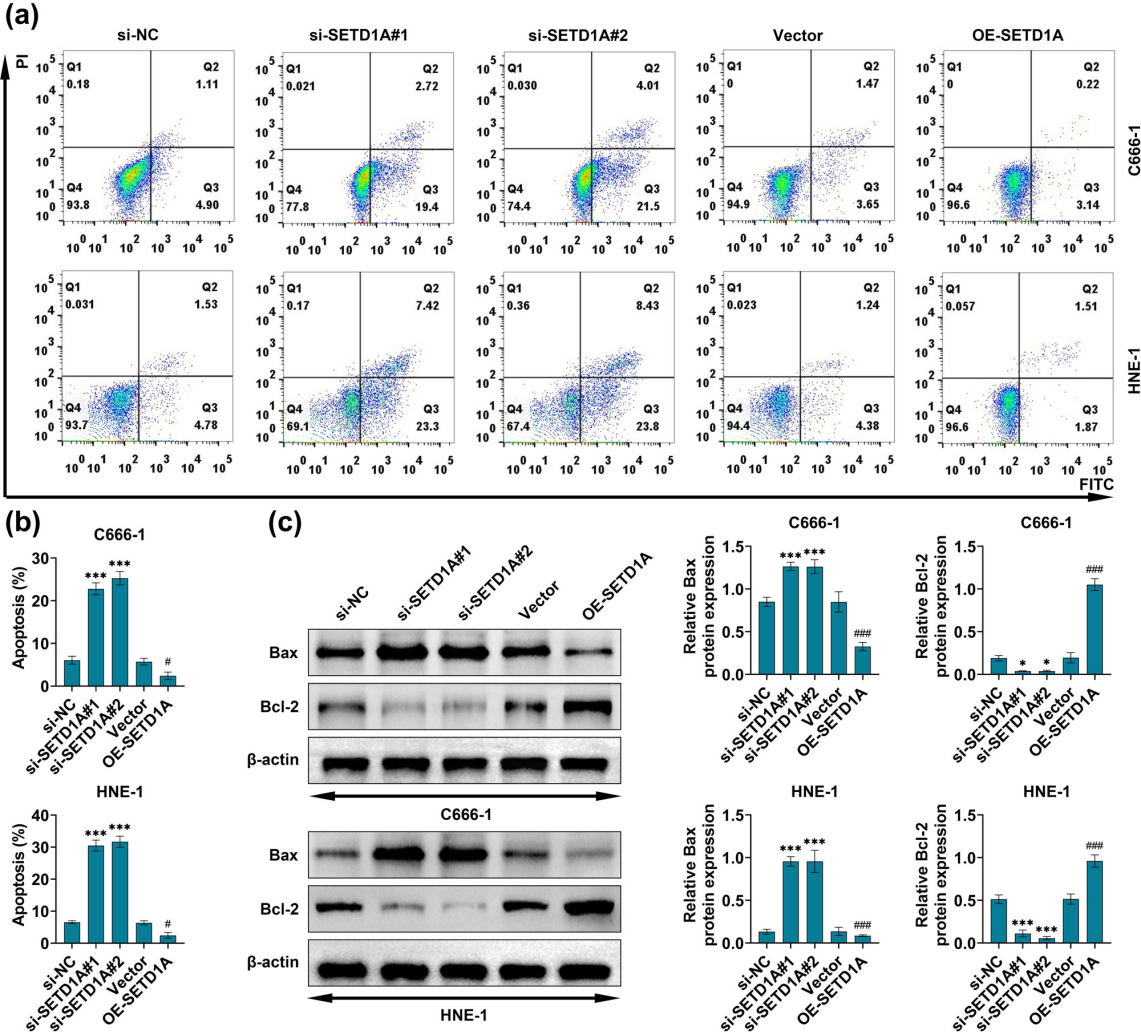
The effects of SETD1A overexpression or knockdown on cellular apoptosis in nasopharyngeal carcinoma cells. (a and b) The flow cytometry analysis was used to measure the effects of SETD1A expression on cellular apoptosis. (c) Western blot was used to explore the protein expression level of Bax and Bcl-2. *p < 0.05 vs si-NC. ***p < 0.005 vs si-NC. ^#^ *p* < 0.05 vs vector. ^###^ *p* < 0.005 vs vector. Data are expressed as mean ± SEM.

To investigate the potential functions of SETD1A in cellular apoptosis of nasopharyngeal carcinoma cells, the cells were transfected with the overexpression or downregulation of SETD1A and the apoptosis rate in different groups was detected by the flow cytometry analysis. As shown in Figure 4a and b, the number of apoptosis-positive cells was significantly higher in the SETD1A downregulation groups than in the si-NC group. Furthermore, when SETD1A overexpressing was performed on the C666-1 and HNE-1 cells, the cellular apoptosis rate was strongly suppressed. Similarly, western blot analysis showed that the apoptosis-related protein (Bax) expression was obviously increased with the knockdown of SETD1A, while the expression of Bcl-2 was suppressed. In contrast, overexpression of SETD1A in C666-1 and HNE-1 cells resulted in significantly decreased Bax expression and the expression of Bcl-2 was improved. These results suggested that overexpression of SETD1A contributed to the reduction of the apoptosis in nasopharyngeal carcinoma cells.

### 3.5 SETD1A promotes the progression of nasopharyngeal carcinoma through PI3K/AKT pathway

**Figure 5:**
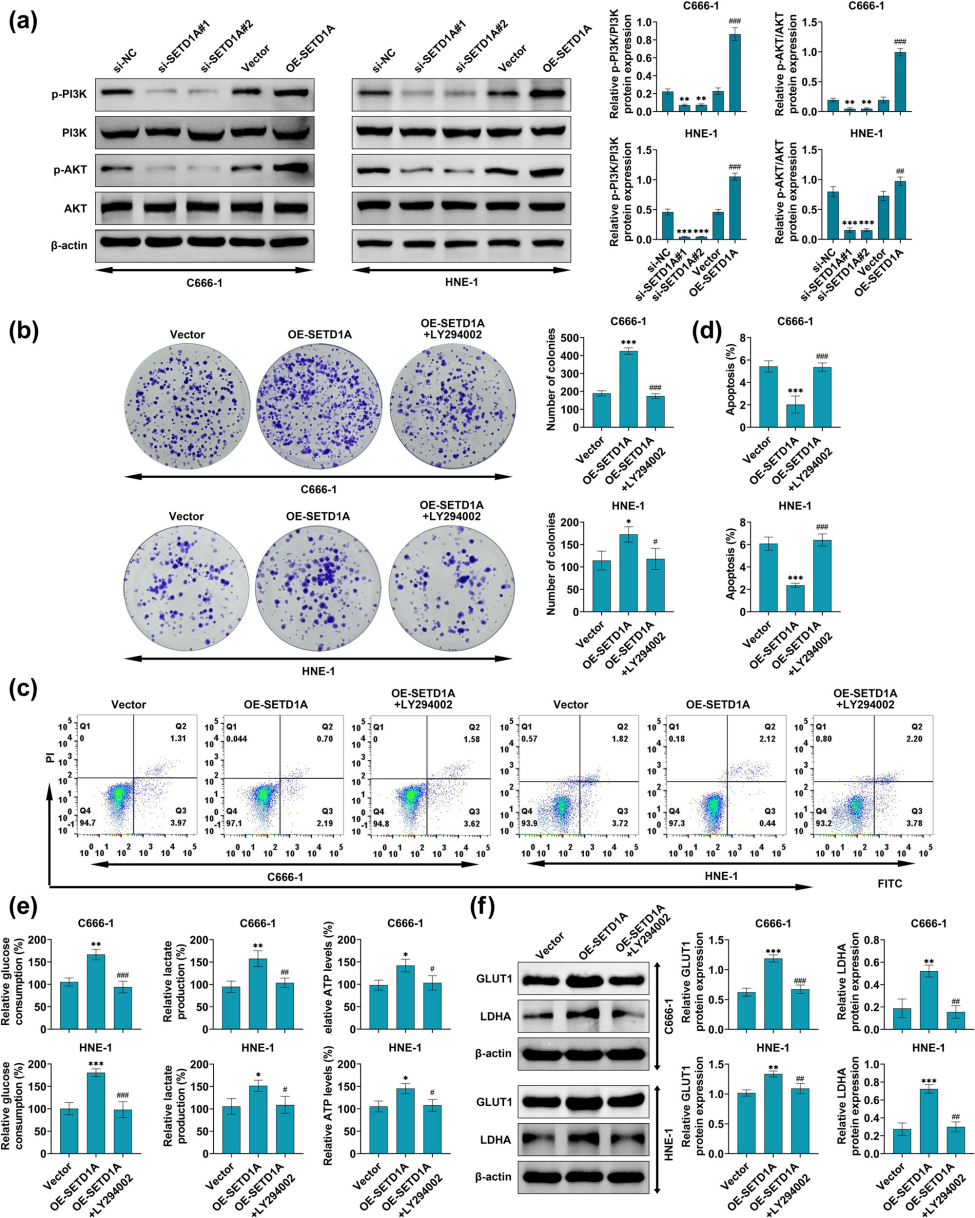
SETD1A is positively regulated nasopharyngeal carcinoma progression via PI3K/AKT pathway. (a) Western blot was used to explore the protein expression level of p-PI3K and p-AKT after si-NC, si-SETD1A#1, si-SETD1A#2, vector, or OE-SETD1A transfected. (b) The colony-forming assay was used to measure the effects of SETD1A expression on cell proliferation after LY294002 treatment. (c, d, and f) The flow cytometry analysis was used to measure the cellular apoptosis after LY294002 treatment. (e) Detection of glucose consumption, lactate production, and ATP levels in C666-1 and HNE-1 cells using commercial kits after LY294002 treatment. (f) Western blot was used to explore the protein expression level of GLUT1 and LDHA. *p < 0.05 vs si-NC. **p < 0.01 vs si-NC. ***p < 0.005 vs si-NC. ^#^ *p* < 0.05 vs vector. ^##^ *p* < 0.01 vs vector. ^###^ *p* < 0.005 vs vector. Data are expressed as mean ± SEM.

To clarify the potential mechanism constituting by which SETD1A promotes the progression of nasopharyngeal carcinoma, PI3K/AKT signaling pathway expression was measured by western blot. The knockdown of SETD1A remarkably suppressed the activation of PI3K and AKT; conversely, in the SETD1A overexpressing group, the activation of PI3K and AKT was raised (Figure 5a). To verify these results, the PI3K inhibitor LY294002 was performed to co-treatment with OE-SETD1A in C666-1 and HNE-1 cells. The results from the clone formation assay indicated that co-treatment with LY294002 in SETD1A overexpression group performed the inhibition of proliferative (Figure 5b). Similar results were observed in the apoptosis assay; when LY294002 was co-treated with OE-SETD1A, the cellular apoptosis rate was significantly increased (Figure 5c and d). Moreover, this study also found that when LY294002 was co-treated with OE-SETD1A, the effect of SETD1A on cellular glucose uptake, lactate production, and ATP levels was obviously attenuated (Figure 5e). Similar results were observed in the western blot assay, when LY294002 was co-treated with OE-SETD1A, the OE-SETD1A-induced LDHA and GLUT1 overexpression were significantly reduced (Figure 5f). These results suggest that SETD1A promotes the progression of nasopharyngeal carcinoma via PI3K/AKT pathway.

## 4 Discussion

Epigenetically altered tumor progenitor genes were employed as pivotal mediators to enhance the likelihood of cancer development and drug resistance [18]. Previous studies have demonstrated that aberrant histone lysine methylation-controlled regulatory induces oncogene expression and frequently contributes to numerous tumorigenesis [19]. In addition, the interfere with transcription factor lysine methylation could suppress the viability of cancer cells. SETD1A is involved in the epigenetic regulation of transcription containing histone lysine methyltransferase, which highly involved in cancer progression. Recent studies reported that SETD1A was highly expressed in hepatocellular carcinoma and breast cancer cells while high SETD1A results in sorafenib resistance [15,20]. However, the role of SETD1A in nasopharyngeal carcinoma remains unclear. The pathogenesis of nasopharyngeal carcinoma has not yet been completely defined. Moreover, the long-term prognosis of nasopharyngeal carcinoma is still unsatisfactory. Changing or modifying the disease-related genes or their expression has become a central issue in the biological treatment of nasopharyngeal carcinoma. A lot of signaling pathways have been found to be related to the molecular mechanism of nasopharyngeal carcinogeneses, such as Wnt/β-catenin or PI3K/AKT signaling [21,22]. Here, this study revealed the function of SETD1A in nasopharyngeal carcinoma that the expression of SETD1A was associated with nasopharyngeal carcinoma cells. Silence of SETD1A results in nasopharyngeal carcinoma cell proliferation inhibition and cell apoptosis. By contrast, upregulated SETD1A significantly enhanced the cell proliferation and reduced the apoptosis ratio in nasopharyngeal carcinoma cells. In numerous cancer cells, the aerobic glycolysis is frequently over-activated. The overexpressed glycolysis is necessary for cancer cells’ metastasis and proliferation to supply proteins, lipids, nucleotides’ production, metabolic intermediates, and ATP generation. The conventional anticancer drugs often suppress cancer cell growth by blocking the glycolysis process in cancer cells [23]. The finding in this study consists of the above-mentioned anticancer mechanism; the results indicated that SETD1A remarkably enhances glucose metabolism by upregulating the expression of glycolytic-related proteins. These data imply that knockdown of SETD1A-reduced glycolysis contributes to metabolic stress and further induces the initiation of the cellular apoptosis process in nasopharyngeal carcinoma cells. PI3K/AKT signaling pathway has been involved in the mediation of cancer cell metabolism via multiple mechanisms. Previous studies have reported that over-activation of PI3K/AKT plays a major role in glycolysis promotion in many cancer cells [24,25]. Multiple evidence indicated that PI3K/AKT signaling pathway could be triggered by reactive oxygen species through blocked some protein phosphatase including protein phosphatase 2A and phosphatase and tensin homolog [26,27].

The whole-exome sequencing reported that PI3K signaling is the prominently activated pathway in nasopharyngeal carcinoma; these results revealed that the nasopharyngeal carcinoma oncogenesis may be associated with the activation of PI3K signaling. All these findings indicated that PI3K/AKT signaling pathway activation aberrantly enhances tumorigenesis in nasopharyngeal carcinoma. Nevertheless, the mechanisms of PI3K/AKT activation in biological function of nasopharyngeal carcinoma have not been fully understanded.

This report is the first to link the SETD1A to PI3K/AKT pathway in nasopharyngeal carcinoma cells to the best of our knowledge. Mechanistically, this study used si-NC, si-SETD1A#1, si-SETD1A#2, vector, or OE-SETD1A to knockdown or overexpress the SETD1A in nasopharyngeal carcinoma cells. To explore whether the regulation of cell proliferation, glycolysis, and apoptosis by SETD1A depends on PI3K/AKT signaling. We used LY294002 to inhibit PI3K/AKT signaling in the nasopharyngeal carcinoma cells. This study found that when LY294002 was co-treated with SETD1A overexpression, the cellular apoptosis rate was significantly increased and the SETD1A-induced cellular ATP levels, glucose uptake, glycolysis, and lactate production were obviously attenuated. Therefore, we conclude that SETD1A promoting the progression of nasopharyngeal carcinoma is mainly depended on the PI3K/AKT signaling activation. The previous study indicated that microRNA-21 expression is triggered by LMP1 via the PI3K/Akt/FOXO3a pathway and at last results in chemo-resistance in nasopharyngeal carcinoma cells [28]. However, in this study, the correlation between SETD1A, PI3K/Akt/FOXO3a pathway, and chemo-resistance in nasopharyngeal carcinoma has not been explored yet, which will be the direction of future studies.

## 5 Conclusion

In conclusion, this study identified overexpressed SETD1A as a positive regulator of proliferation that induced nasopharyngeal carcinoma cells’ aerobic glycolysis via PI3K/AKT signaling activation in vitro. This study laid a strong foundation for unveiling the precise anticancer mechanism of SETD1A. In particular, the SETD1A may become a novel target for further inhibitor design to interfere with PI3K/AKT-dependent nasopharyngeal carcinoma progression.

## References

[1] Ngan H-L, Wang L, Lo K-W, Lui VWY. Genomic landscapes of EBV-associated nasopharyngeal carcinoma vs HPV-associated head and neck cancer. Cancers. 2018;10(7):210. 10.3390/cancers10070210 PMC6070978 29933636

[2] Fan C, Tu C, Qi P, Guo C, Xiang B, Zhou M, et al. GPC6 promotes cell proliferation, migration, and invasion in nasopharyngeal carcinoma. J Cancer. 2019;10(17):3926. 10.7150/jca.31345 PMC6692608 31417636

[3] Ben-Ami T, Ash S, Ben-Harosh M, Gavriel H, Weintraub M, Revel-Vilk S, et al. Nasopharyngeal carcinoma in children and young adults—Beyond 5-year survival. Pediatr Blood Cancer. 2020;67(9):e28494. 10.1002/pbc.28494 32573923

[4] Lang J, Hu C, Lu T, Pan J, Lin T. Chinese expert consensus on diagnosis and treatment of nasopharyngeal carcinoma: Evidence from current practice and future perspectives. Cancer Manag Res. 2019;11:6365. 10.2147/CMAR.S197544 PMC6628961 31372041

[5] Liu GY, Lv X, Wu YS, Mao MJ, Ye YF, Yu YH, et al. Effect of induction chemotherapy with cisplatin, fluorouracil, with or without taxane on locoregionally advanced nasopharyngeal carcinoma: A retrospective, propensity score-matched analysis. Cancer Commun. 2018;38(1):1–10. 10.1186/s40880-018-0283-2 PMC5993041 29764487

[6] Spencer NY, Stanton RC. The Warburg effect, lactate, and nearly a century of trying to cure cancer. Semin Nephrol. 2019;39(4):380–93. 10.1016/j.semnephrol.2019.04.007 31300093

[7] Sun L, Suo C, Li ST, Zhang H, Gao P. Metabolic reprogramming for cancer cells and their microenvironment: Beyond the Warburg Effect. Biochim Biophys Acta (BBA)-Rev Cancer. 2018;1870(1):51–66. 10.1016/j.bbcan.2018.06.005 29959989

[8] Fan T, Sun G, Sun X, Zhao L, Zhong R, Peng Y. Tumor energy metabolism and potential of 3-bromopyruvate as an inhibitor of aerobic glycolysis: implications in tumor treatment. Cancers. 2019;11(3):317. 10.3390/cancers11030317 PMC6468516 30845728

[9] Rascio F, Spadaccino F, Rocchetti MT, Castellano G, Stallone G, Netti GS, et al. The pathogenic role of PI3K/AKT pathway in cancer onset and drug resistance: An updated review. Cancers. 2021;13(16):3949. 10.3390/cancers13163949 PMC8394096 34439105

[10] Zou Z, Tao T, Li H, Zhu X. mTOR signaling pathway and mTOR inhibitors in cancer: Progress and challenges. Cell Biosci. 2020;10(1):1–11. 10.1186/s13578-020-00396-1 PMC7063815 32175074

[11] Ren JH, Hu JL, Cheng ST, Yu HB, Wong VKW, Law BYK, et al. SIRT3 restricts hepatitis B virus transcription and replication through epigenetic regulation of covalently closed circular DNA involving suppressor of variegation 3-9 homolog 1 and SET domain containing 1A histone methyltransferases. Hepatology. 2018;68(4):1260–76. 10.1002/hep.29912 29624717

[12] Matsui H, Iriyama T, Sayama S, Inaoka N, Suzuki K, Yoshikawa M, et al. Elevated placental histone H3K4 methylation via upregulated histone methyltransferases SETD1A and SMYD3 in preeclampsia and its possible involvement in hypoxia-induced pathophysiological process. Placenta. 2021;115:60–9. 10.1016/j.placenta.2021.09.009 34560329

[13] Du M, Gong P, Zhang Y, Liu Y, Liu X, Zhang F, et al. Histone methyltransferase SETD1A participates in lung cancer progression. Thorac Cancer. 2021;12(16):2247–57. 10.1111/1759-7714.14065 PMC8365002 34219384

[14] Kang J-Y, Park JW, Hwang Y, Hahm JY, Park J, Park K-S, et al. The H3K4 methyltransferase SETD1A is required for proliferation of non-small cell lung cancer cells by promoting S-phase progression. Biochem Biophys Res Commun. 2021;561:120–7. 10.1016/j.bbrc.2021.05.026 34023776

[15] Wu J, Chai H, Li F, Ren Q, Gu Y. SETD1A augments sorafenib primary resistance via activating YAP in hepatocellular carcinoma. Life Sci. 2020;260:118406. 10.1016/j.lfs.2020.118406 32918976

[16] Wu J, Chai H, Xu X, Yu J, Gu Y. Histone methyltransferase SETD1A interacts with HIF1α to enhance glycolysis and promote cancer progression in gastric cancer. Mol Oncol. 2020;14(6):1397–409. 10.1002/1878-0261.12689 PMC7266269 32291851

[17] Li S, Jia H, Zhang Z, Wu D. LncRNA GAS6-AS1 facilitates the progression of breast cancer by targeting the miR-324-3p/SETD1A axis to activate the PI3K/AKT pathway. Eur J Cell Biol. 2020;99(8):151124. 10.1016/j.ejcb.2020.151124 33223203

[18] Shah K, Rawal RM. Genetic and epigenetic modulation of drug resistance in cancer: challenges and opportunities. Curr Drug Metab. 2019;20(14):1114–31. 10.2174/1389200221666200103111539 31902353

[19] McGrath J, Trojer P. Targeting histone lysine methylation in cancer. Pharmacol Ther. 2015;150:1–22. 10.1016/j.pharmthera.2015.01.002 25578037

[20] Jin ML, Kim YW, Jin HL, Kang H, Lee EK, Stallcup MR, et al. Aberrant expression of SETD1A promotes survival and migration of estrogen receptor α-positive breast cancer cells. Int J Cancer. 2018;143(11):2871–83. 10.1002/ijc.31853 PMC6278950 30191958

[21] Xiang S, Xiang T, Xiao Q, Li Y, Shao B, Luo T. Zinc-finger protein 545 is inactivated due to promoter methylation and functions as a tumor suppressor through the Wnt/β-catenin, PI3K/AKT and MAPK/ERK signaling pathways in colorectal cancer. Int J Oncol. 2017;51(3):801–11. 10.3892/ijo.2017.4064 PMC5564408 28677721

[22] Peng L, Huang Y-T, Zhang F, Chen J-Y, Huo X. Chronic cadmium exposure aggravates malignant phenotypes of nasopharyngeal carcinoma by activating the Wnt/β-catenin signaling pathway via hypermethylation of the casein kinase 1α promoter. Cancer Manag Res. 2019;11:81. 10.2147/CMAR.S171200 PMC6304082 30588112

[23] Pelicano H, Martin D, Xu R, Huang P. Glycolysis inhibition for anticancer treatment. Oncogene. 2006;25(34):4633–46. 10.1038/sj.onc.1209597 16892078

[24] Yang J, Nie J, Ma X, Wei Y, Peng Y, Wei X. Targeting PI3K in cancer: mechanisms and advances in clinical trials. Mol Cancer. 2019;18(1):1–28. 10.1186/s12943-019-0954-x PMC6379961 30782187

[25] Vadlakonda L, Pasupuleti M, Pallu R. Role of PI3K-AKT-mTOR and Wnt signaling pathways in transition of G1-S phase of cell cycle in cancer cells. Front Oncol. 2013;3:85. 10.3389/fonc.2013.00085 PMC3624606 23596569

[26] Chen L, Xu B, Liu L, Luo Y, Zhou H, Chen W, et al. Cadmium induction of reactive oxygen species activates the mTOR pathway, leading to neuronal cell death. Free Radic Biol Med. 2011;50(5):624–32. 10.1016/j.freeradbiomed.2010.12.032 PMC3032035 21195169

[27] Wu H, Goel V, Haluska FG. PTEN signaling pathways in melanoma. Oncogene. 2003;22(20):3113–22. 10.1038/sj.onc.1206451 12789288

[28] Zhang H, Wang J, Yu D, Liu Y, Xue K, Zhao X. Role of Epstein-Barr virus in the development of nasopharyngeal carcinoma. Open Med. 2017;12(1):171–6. 10.1515/med-2017-0025 PMC5471915 28730175

